# EEG-Based Emotion Classification Using Long Short-Term Memory Network with Attention Mechanism

**DOI:** 10.3390/s20236727

**Published:** 2020-11-25

**Authors:** Youmin Kim, Ahyoung Choi

**Affiliations:** Department of Software, Gachon University, Seongnam 13120, Korea; ymkim@emro.co.kr

**Keywords:** EEG, emotion classification, deep learning, long short-term memory

## Abstract

Recently, studies that analyze emotions based on physiological signals, such as electroencephalogram (EEG), by applying a deep learning algorithm have been actively conducted. However, the study of sequence modeling considering the change of emotional signals over time has not been fully investigated. To consider long-term interaction of emotion, in this study, we propose a long short-term memory network to consider changes in emotion over time and apply an attention mechanism to assign weights to the emotional states appearing at specific moments based on the peak–end rule in psychology. We used 32-channel EEG data from the DEAP database. Two-level (low and high) and three-level (low, middle, and high) classification experiments were performed on the valence and arousal emotion models. The results show accuracies of 90.1% and 87.9% using the two-level classification for the valence and arousal models with four-fold cross validation, respectively. In the case of the three-level classification, these values were obtained as 83.5% and 82.6%, respectively. Additional experiments were conducted using a network combining a convolutional neural network (CNN) submodule with the proposed model. The obtained results showed accuracies of 90.1% and 88.3% in the case of the two-level classification and 86.9% and 84.1% in the case of the three-level classification for the valence and arousal models with four-fold cross validation, respectively. In 10-fold cross validation, there were 91.8% for valence and 91.6% for arousal accuracy, respectively.

## 1. Introduction

According to the recent announcement by the WHO, 264 million people worldwide suffer from depression [1]. Emotion analysis is necessary for patients with depression. Many studies have been conducted on emotion analysis based on users’ facial expression, behavior recognition, and physiological signals, such as electrocardiogram (ECG), electromyography (EMG), and electroencephalogram (EEG) signals [2,3]. However, emotion recognition through facial expressions or behavior analyzes the emotions expressed and not the naturally aroused emotions. Internally aroused emotions are induced through autonomic nervous system responses or the degree of activation of the parts of the brain that communicate emotions. Therefore, research on recognizing emotions through brain signals, such as Functional magnetic resonance imaging (fMRI), EEG, positron emission tomography (PET) and so on, is being actively conducted [4,5,6].

Among them, studies on emotion recognition based on EEG signals have been actively conducted because EEG signals can be measured noninvasively. Researchers have conducted studies to extract features from EEG signals and apply machine learning and deep learning techniques to analyze emotion using two-level (high and low) or three-level (low, middle, and high) classification methods based on valence and arousal emotion models [7,8,9,10,11]. Al-Fahoum et al. proposed an EEG signal-based emotion recognition methodology that extracts features based on fast Fourier and wavelet transforms by using eigenvector-based and autoregressive methods; the extracted features are classified into a specific emotion model. Posner et al. proposed a valence–arousal model consisting of valence, representing emotion positiveness, and arousal, representing emotional strength [12]. Chung and Yoon used 32 EEG channels from the DEAP database and 61 virtual EEG channels created by the bipolar montage technique [7]. They extracted power spectral density and power asymmetry from the signal and used the Bayes classifier to classify the valence and arousal into two classes (high or low). The average classification result was 53.4% for the two-level valence and arousal recognition model. Koelstra et al. proposed a method to extract the power spectral density and power asymmetry from EEG signals, and the valence and arousal spaces were classified as high or low by using the naïve Bayes classifier [8]. The results showed an accuracy of 57.0% for valence and 62.0% for arousal. Zhang et al. proposed ontological models by extracting process structure diagrams (PSDs) from the DEAP database [9]. The accuracy was 75.19% in valence and 81.74% in arousal. Atkinson and Campos proposed a method that extracts the features of statistical characteristic values, band power, Hjorth parameters, and fractal dimensions from the DEAP database by selecting 14 EEG channels [10]. They trained a support vector machine (SVM) using these features as input, and the results showed classifications of 73.14% and 73.06% for the valence and arousal classes. Al-Nafjan et al. extracted PSD and frontal asymmetry from the EEG channel and classified them into four emotional states, namely meditation, boredom, excitement, and frustration under valence, and the arousal dimension by applying a deep neural network [13]. Each class showed an accuracy of 82.0%. Krishna et al. proposed an emotion recognition method based on a generalized mixture model (GMM) to extract symmetrical or asymmetrical EEG signals using a brain wave (EEG) signal dataset. It was confirmed that it was very useful to recognize the signal of the EEG sample in a short period, and the average accuracy was 89.1% [14].

The existing studies used a method of analyzing emotion by inputting specific feature values or patterns obtained from the EEG signal. However, it is difficult to use in general because the features found differ depending on the researcher or the database. Therefore, research on an end-to-end model that minimizes preprocessing such as feature extraction is needed. Karim et al. proposed a multivariate long short-term memory (LSTM) network and a convolutional neural network (CNN) model and verifies the effectiveness of the proposed algorithm using 35 publicity available databases without a feature extraction process [15]. In particular, they found that squeeze and excitation block improved the accuracy of recognition. Jirayucharoensak et al. proposed a deep learning network with stacked AutoEncoder as functional learning by inputting the power spectrum density of nonstationary EEG signals [11]. The accuracy of the proposed method was 49.02% in the valence class and 46.03% in the arousal class for three-level classification. Yang et al. proposed a multi-column structured CNN model using the raw signal of EEG as an input [16]. The model was constructed using four convolutional layers and one fully connected layer, and two-level classification was performed using the DEAP database. The valence performance was 90.01% and the arousal performance was 90.65%.

In recent years, since the EEG signals change over time and are particularly affected by a previous state or situation, many studies have been conducted that analyzed the trend of emotion over time. In many neuroimaging studies, dynamics of emotional experiences have been studied [17,18,19]. They found that when emotions change over time, neural networks of the medial prefrontal cortex, amygdala, and insula were involved [17,18]. Resibois et al. especially demonstrated that the neural basis of emotion changes with time through two ganglion processes. At the beginning, the explosiveness or steepness of the emotional reaction is reflected, and the accumulation and reinforcement of the reaction to the emotion appears [19]. Emotion accumulation was positively associated with bilateral activation of the insula and target cortex, right carapace and anterior target cortex, and left mid-frontal area, while emotional explosiveness was positively associated with activity of the left medial prefrontal cortex (mPFC) and right cerebellum. Therefore, through existing studies, it can be seen that changes in emotion over time affect neurological brain activation, and these changes can appear in EEG signals over time. Zheng et al. investigated whether there is a pattern that appears to be stable over time [20]. They verified that significant results were obtained when the same session was conducted on different days. Power spectral density (PSD), differential entropy (DE), differential asymmetry (DASM), rational asymmetry (RASM), asymmetry (ASM), and differential caudality (DCAU) features from the EEG over time graph regularized the extreme learning machine method. As a result of the experiment, it was reported that the relationship between the change in emotional state and the EEG signal was recognized stably with an accuracy of 79.28% to one person for a certain period of time. For classification of time-series data, the recurrent neural network (RNN) model and long short-term memory network (LSTM) model were designed to consider that the current information may be affected by the previous state to solve the long-term dependency problem [21]. Huang et al. suggested a convolutional recurrent network that estimate the emotion from speech signals [22]. They found that time information must be included in the model for speech-based emotion recognition, and that the model using the CNN model was robust against noise. Alhagry et al. proposed an LSTM network for two-level classification from raw EEG signals [23]. They confirmed that the accuracy of the valence was 85.4% and that of the arousal was 85.6% in two-level classification. Anubhav et al. proposed an LSTM-based emotion recognition approach that calculates the band power of the EEG signal and inputs the frequency characteristics [24]. The DEAP dataset was applied to the model, and the accuracy was 94.69% for valence recognition and 93.13% for arousal recognition in two-level classification. Xing et al. presented a framework consisting of a linear EEG mixing model designed using Stack AutoEncoder and an emotional timing model using LSTM-RNN [25]. Their framework decomposed the EEG source signal from the collected EEG signal and used context correlation of EEG functional sequences to improve classification accuracy. Their model achieved 81.10% accuracy for valence and 74.38% for arousal in the DEAP dataset.

Therefore, in this study, we propose an end-to-end LSTM model in order to recognize emotions that change over time without feature extraction of EEG signals. We use a time-series raw EEG signal as an input without feature extraction and applied the LSTM network to consider the emotional signal change over time. In addition, we apply the attention mechanism to implement the psychological theory, the peak–end rule, according to which a certain state among the previous states greatly affects the current emotion [26]. The attention mechanism is a function used in the field of natural language processing that focuses on the part of the input word that is related to the word to be predicted out of the entire input sentence from the encoder at every time step when the decoder predicts the output word [27]. In this work, an attention weight is calculated to determine which part of the input has the most influence on the output, and the higher the weight of the corresponding input part, the larger is the value in the network training. In addition, many existing studies have been performed on feature extraction based on a CNN model. To improve the accuracy of emotion recognition, we propose a hierarchical model that applies CNN to the LSTM model with an attention mechanism. We observe that the proposed method minimizes information loss that may occur in the feature extraction step and evaluate the recognition accuracy in two-level emotion classification and three-level emotion classification.

The main contributions of this paper are as follows: First, we are the first to propose an attention mechanism to an LSTM model for EEG-based emotion recognition to improve emotion recognition accuracy by considering psychological theory, the peak–end rule. Second, we are the first to propose a hierarchical model that applies CNN to the attention LSTM model in parallel to ensure robust recognition even when the number of classes in emotion analysis increases. Third, we propose an end-to-end emotion analysis model while minimizing the preprocessing step to guarantee the emotion recognition accuracy in general. The next sections are described as follows. Section 2 indicates the dataset, the architecture, and equations of the proposed methods and Section 3 shows the experimental result. In Section 4, we discuss the experimental result and limitations of this work. Then we conclude the manuscript in Section 5.

## 2. Materials and Methods

### 2.1. Dataset

In this study, we used the DEAP database for obtaining the input data [8]. This database comprises information about 32 participants with an average age of 26.9 years, who watched a 1 min music highlight video in various genres such as sadness, pleasure, satisfaction, interest, and anger. A total of 120 1 min music highlight videos were selected in this study. The selected scenes were randomly given to 40 online participants, and the subjective feeling conveyed from the video was recorded based on the self-assessment manikin (SAM) questionnaire. A nine-point scale was used to measure the emotions of valence, arousal, dominance, liking, and familiarity. While the subjects watched the music video, their EEG signals were measured using an EEG-measuring device with 32 electrodes on the scalp. Thus, the DEAP database comprised 32-channel EEG signals of subjects and 12 peripheral channels including EMG, skin conductance, and respiration; three unused channels; and one status channel. In this study, we used the 32-channel EEG signals as input data. Pretreatment was applied to each EEG signal. The EEG measured at 512 Hz was downsampled to 128 Hz, and the baseline of each datum was reduced to 3 s. The data about the Fp1 and Fp2 channels were filtered by electrooculography (EOG) signals because some values from these Fp1 and Fp2 channels were affected by the value of the eye blinking and the movement of the pupils. To remove noise from other channels, including the corresponding channel, a bandwidth filter with a frequency range of 4.0–45.0 Hz, which is generally used in brain waves, was applied.

**Figure 1:**
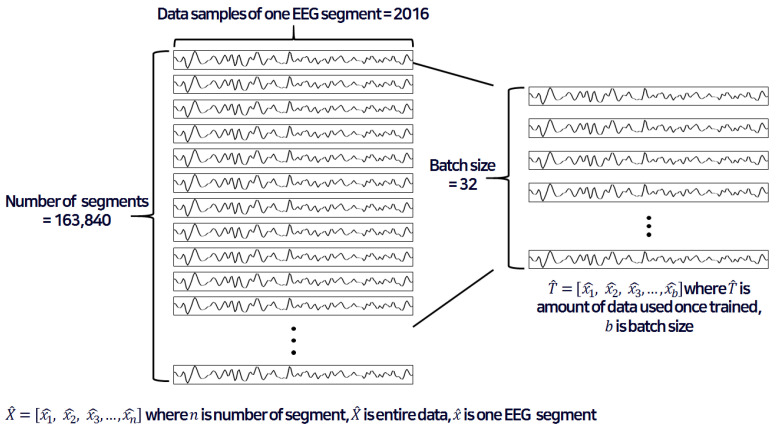
Example of an electroencephalogram (EEG) input segment with a window of about 15 s (exactly 15.75 s).

Figure 1 shows input data to be used in the network for training. $\hat{X}$ represents the entire dataset that is 32-channel EEG signals of all subjects and $\hat{x}$ refers to the one EEG segment that refers to a block of EEG signals divided by the window size. The EEG signals are segmented as input into the deep learning network. To recognize the change in emotion over time, this study analyzed the results by segmenting the input signal into a certain time interval. We tested 5.25-, 15.75-, and 30.50-second windows to find the optimal size of input signal in Section 3.1. When one EEG signal acquired in one session is divided by 12, one segment contains approximately 5 s (exactly 5.25 s) of data. Likewise, dividing one EEG signal by 4 contains about 15 s of data (exactly 15.75 s) per one segment, and dividing by 2 contains about 30 s of data (exactly 31.50 s) per one segment. As a result, we observed the best performance when we divided the entire data into 15.75 s windows to form one segment. In addition, the amount of data used for training is $\hat{T}$ and the batch size is denoted as b. Batch refers to the number of inputs used when learning once in the network. In this study, the number of EEG channels is 32, so the batch size was designated as 32. 

The data measured in one EEG channel include a 3 s baseline EEG signal and 60 s EEG signal while watching a 1 min movie clip. The reason for including the baseline as an input is to detect the amount of physiological change with certain stimuli, as indicated by Lang et al. [6]. The sampling frequency is 128 Hz, so there are 8064 data samples for a total of 63 s in a one-channel EEG signal. Then we divided this data into four segments with each segment containing a 15.75 s long signal with 2016 data samples per segment. There are a total of 330,301,440 data points under the condition of 32 subjects × 32 EEG channels × 40 experiments × 8064 data points per segment. We get 163,840 segments since 330,301,440 data points are divided by 2016 data points. Thus, 163,840 entire segments are used for training and testing in this study. Output data were used based on the valence and arousal levels. For discrete emotion recognition, we classify the arousal and valance according to two-level (low and high classes) and three-level (low, medium, and high) classifications. The numbers of data points for the low and high classes were 59,840 and 104,000, respectively, in the two-level classification. In the case of the three-level classification, 20,160, 60,160, and 83,520 data points were considered for low, medium, and high classes.

### 2.2. Emotion Classificiation Using LSTM Model and Attention Mechanism

To solve the long-term dependency problem in the recurrent neural network model developed to influence the previous learning information in the future, we applied the LSTM model proposed by Hochreiter and Schmidhuber to analyze emotions [21]. Accordingly, we could design a network in which not only the emotions currently being felt but also the emotions felt before are considered. In addition, we apply the attention mechanism to implement the psychological theory, the peak–end rule, according to which a certain state among the previous states greatly affects the current emotion [27]. The attention mechanism is a function used in the field of natural language processing that focuses on the part of the input word that is related to the word to be predicted out of the entire input sentence from the encoder at every time step when the decoder predicts the output word. In recent years, many studies, such as gear fault diagnosis from acoustic signals [28], object recognition [29], and visual question answering [30], reported improved accuracy by integrating the attention mechanism with the deep learning algorithm. In addition, many studies of speech signal-based emotion recognition have been conducted that apply attention techniques to recurrent neural networks or convolutional neural networks [20,31,32]. However, the method of applying the attention mechanism for EEG signal-based emotion recognition, where the relationship between the signals before and after is not clear, has not been fully investigated yet. In this work, an attention weight is calculated to determine which part of the input has the most influence on the output, and the higher the weight of the corresponding input part, the larger is the value in the network training.

**Figure 2:**
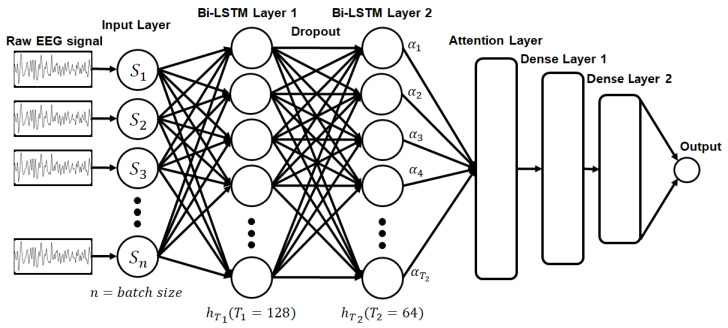
Proposed long short-term memory (LSTM) network with attention mechanism.

Figure 2 displays the proposed network that consists of two bidirectional LSTM layers, an attention layer to apply the attention mechanism, and two density layers to produce the final output. The reason for using bidirectional LSTM is because it is known that not only the emotional signal is affected by the previous signal, but also the stimulation is transmitted by the autonomic nervous system response for several seconds after the stimulation occurs, so it was applied to consider the time step before and after [30]. The first hidden layer uses a bidirectional LSTM layer with 128 neurons and a rectified linear unit (ReLU) as the activation function. Then, to prevent the overfitting problem, a dropout with a probability of 0.2 is executed and passed through the second bidirectional LSTM layer with 64 neurons and a ReLU as the activation function. After calculating the attention weight with the hidden state, which is the output of the second bidirectional layer, the output dimension with size 16 is reduced by dense layer 1 by using the ReLU. Finally, we achieve an output size of 1, and the final output of the two-level classification is calculated by passing dense layer 2 using sigmoid as the activation function. In the three-level classification, the output size is 3, and the final output is calculated using softmax as the activation function. The final output for valence and arousal using the two-level classification ranged from 1 to 5 and 5 to 9 for the low and high classes, respectively, and from 1 to 3, 3 to 6, and 6 to 9 for the low, middle, and high classes, respectively, in the case of the three-level classification.

**Figure 3:**
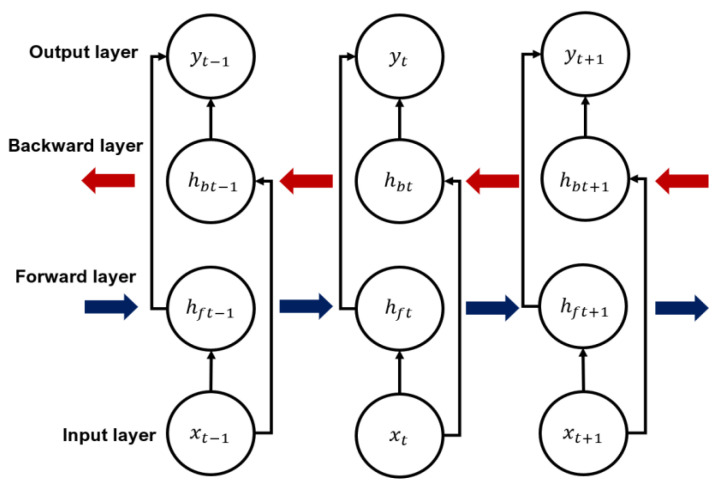
Bidirectional LSTM structure and notation.

The Bi-LSTM model was used in this study and is calculated as follows. The LSTM cell is composed of an input gate, a forget gate, and an output gate, and each calculation method is defined by Equations (1)–(6).

(1) $$i_{t}=\sigma \left(W_{i}\cdot \left[h_{t-1},\;x_{t}\right]+b_{i}\right),$$

(2) $$f_{t}=\sigma \left(W_{f}\cdot \left[h_{t-1},\;x_{t}\right]+b_{f}\right),$$

(3) $$g_{t}=\tan h\left(W_{C}\cdot \left[h_{t-1},\;x_{t}\right]+b_{C}\right),$$

(4) $$C_{t}=f_{t}C_{t-1}+i_{t}g_{t},$$

(5) $$o_{t}=\sigma \left(W_{o}\cdot \left[h_{t-1},\;x_{t}\right]+b_{o}\right),$$

(6) $$h_{t}=o_{t}\tan h\left(C_{t}\right),$$

where *t* is the current time step, $i_{t}$ is the input gate, $f_{t}$ is the forget gate, $g_{t}$ is the cell state, $o_{t}$ is the output gate, and $h_{t}$ is the hidden state of the current cell. W is a weight matrix used in each gate, x is the input, and σ is the sigmoid function. According to the calculation method of the LSTM cell, the bidirectional LSTM structure calculates the hidden state of forward and backward directions, and calculates the output reflecting both [33]. Figure 3 shows the progression structure of the bidirectional LSTM, the calculation method of which is defined by Equations (7)–(9).

(7) $$h_{ft}=\sigma \left(W_{x_{t}}+W_{h_{\mathrm{ft}-1}}+b\right),$$

(8) $$h_{bt}=\sigma \left(W_{x_{t}}+W_{h_{\mathrm{bt}-1}}+b\right),$$

(9) $$y_{t}=W_{h_{ft}}+W_{h_{bt}}+b,$$

where $h_{ft}$ represents the hidden state of the LSTM cell passing in the forward direction and $h_{bt}$ represents the hidden state of the LSTM cell passing in the reverse direction. Final output $y_{t}$ is calculated by multiplying the two hidden states by the weight matrix and then adding the products.

**Figure 4:**
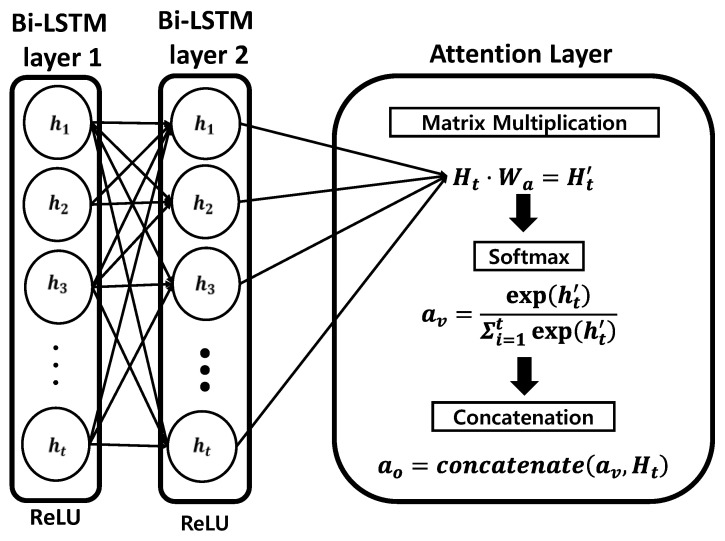
Structure and procedure of calculating an attention layer.

The attention weight calculated as 2 is depicted in Figure 4; it is calculated to determine which part of the input has the most influence on the output. The higher the weight of the corresponding input part, the larger is the value when the network is trained. The calculation procedure is as follows:

(10) $$H_{t}^{\prime }=H_{t}\cdot W_{a},$$

(11) $$a_{v}=\frac{\exp\left(h_{t}^{\prime }\right)}{\sum_{i=1}^{t}\exp\left(h_{i}^{\prime }\right)},$$

(12) $$a_{o}=concatenate\left(a_{v},h_{t}\right).$$

Hidden state $H_{t}$ is calculated by multiplying the second bidirectional LSTM layer with the randomly initialized attention weight, $W_{a}$; the length of the attention weight is the same as the length of hidden state $H_{t}$. The result of this calculation is converted to a probability value through softmax, where h′ is an element of H′ obtained through Equation (10). The transformed attention vector, $a_{v}$, is added to the initially calculated hidden state $H_{t}$ to produce the final attention output, $a_{o}$. Dense layer 1 connected to the attention layer receives attention output $a_{o}$ as an input and reflects the weight of the part that is the most important in future learning to produce more accurate results.

The Adam optimizer was used as the optimization function, and the learning rate was set to 0.001. In addition, a cross-entropy loss function suitable for binary classification was used. Further, a stratified K-fold cross validation method was used to measure the accuracy, with four folds as well as 10 folds. Through the four-fold cross validation method, the labels were distributed in a balanced manner to each fold for learning; 75% of the data were used as the training set and 25% as the test set for training of each fold for four-fold cross validation. For 10-fold cross validation, 90% of the data were used for training and the other data used for testing. In K-fold cross validation, as the number of K decreases, the data used for learning and training are similar and thus the accuracy improves, but it is difficult to generalize because of overfitting problems. Therefore, in this study, we tested both four-fold cross validation as well as 10-fold cross validation to confirm whether validation was subject independent. The train and test data were randomly grouped and generated without subject classification using the data shuffle function. The epoch was set to 30, such that each fold was trained 30 times, and the batch size for input was set to 32. Experiments were conducted to optimize each hyperparameter, as described in in Section 3.1.

### 2.3. Emotion Classificiation by Using Attention LSTM and CNN Model

In this section, we discuss constructing a model for emotion recognition by processing the CNN model in parallel with the attention LSTM model. In general, a CNN model is used for state or object recognition; therefore, in this study, we used the attention LSTM and CNN models to reflect the change of emotion over time to determine any performance improvement in the emotion recognition accuracy. The accuracy of the classification problem could be increased through data preprocessing, feature extraction, or designing the structure so that more diverse features can be considered when the network is trained without additional preprocessing and feature extraction processes. Therefore, we designed a network to increase accuracy by using the previously used input. In our methods, the LSTM + attention network is used as the main module and the CNN network is used as a submodule by rearranging the dimensions of the input to design a network that can consider features using convolutions.

**Figure 5:**
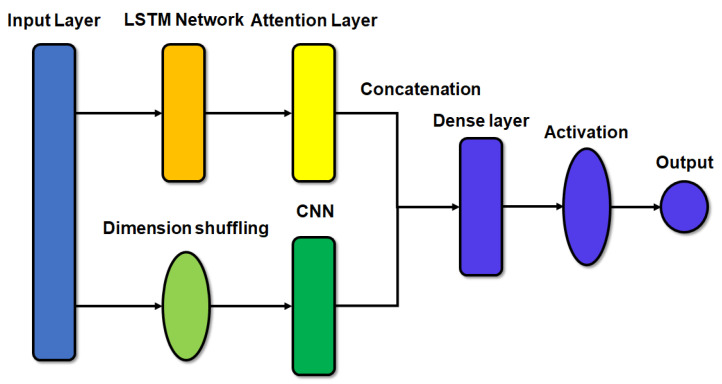
Structure of the LSTM network with attention mechanism and convolutional neural network (CNN) module.

Figure 5 shows the network structure diagram. After receiving the existing input from the main module, the LSTM module passes through the layer by using the attention mechanism. The structure of the LSTM module used here is the same as that described in Section 2.2. In the CNN module (submodule), the process of dimension shuffling changes the dimension of the input. Therefore, the input changes along the *x*- and *y*-axes. The input then passes through the CNN consisting of two convolution layers. The hidden state calculated in each module is combined, passed through the dense layer, and passed through the activation function. In the case of the two-level classification, the activation function had a value between 0 and 1, and it was then rounded to calculate the final value. For the three-level classification, softmax was used to select the class with the highest probability from the three classes.

**Figure 6:**
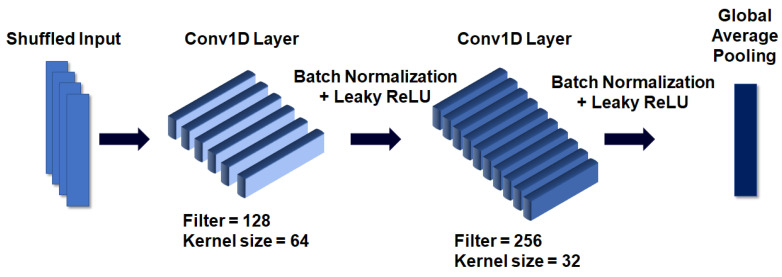
Structure of CNN used as a submodule.

The structure of the CNN submodule, illustrated in Figure 6, is as follows. As mentioned earlier, the input was used after undergoing a dimension shuffle process that changes the *x*- and *y*-axes of the EEG segment. A segment is described as an EEG signal value divided by a window in which the length of the *x*-axis is set, and the *y*-axis is 1 because of the use of a 1-D signal. This input is used as input to a 1-D convolution layer with 128 filters and a kernel size of 64. After batch normalization is applied to the hidden state generated by the operation to solve the overfitting problem, the leaky ReLU activation function is applied. We used the leaky ReLU activation function to prevent the occurrence of the dying ReLU problem due to the formation of negative values by the ReLU activation function as the network progresses. The computed hidden state passes through a second 1-D convolution layer with 256 filters and a kernel size of 32, and then batch normalization and the leaky ReLU activation function are applied to the state. Global average pooling is applied to convert this hidden state into a 1-D state. The final result in this network is used in combination with the result of the main module of the LSTM + attention network.

## 3. Experimental Results

### 3.1. Experimental Setup and Parameter Opimization

The training and testing environment was run on a PC with an Intel (R) Core (TM) i7-6850K CPU @ 3.60 GHz, six cores, 12 logical processors with 64 GB of RAM, and two NVIDIA GeForce GTX 1080Tis. The deep learning model was implemented with Keras, a Python deep learning library. For training and testing, we applied the four-fold cross validation and 10-fold cross validation. The time performance of each validation method was as follows. In the case of four-fold validation, Attention LSTM took about 2 h to train the model and about 20 s to test the model. In detail, it took about 30 s per 1 epoch, and one-fold validation was made up of 30 epochs, so it took about 2 h after turning all four folds. For attention + LSTM + CNN, it took about 4 min per one epoch, and 2 h for 30 epochs. Overall, it took about 8 h after turning all four folds. In the case of 10-fold cross validation, Attention LSTM took about 30 s for one epoch, 25 min for one-fold validation for 50 epochs, and 4 h for 10-fold validation. attention + LSTM + CNN took about 5 min 30 s for one epoch, about 4 h 40 min for one-fold validation based on 50 epochs, and about 46 h for 10-fold validation. In summary, in the Attention LSTM model, there was no difference in the training time of 10-fold validation and the training time of four-fold validation. In the Attention LSTM + CNN model, when validating with 10-fold, it took about 10 times longer than four-fold cross validation.

Optimization was performed for the training of the deep learning model. In this experiment, the number of cells in the LSTM layer, number of LSTM layers, and length of the EEG segment were included in the optimization. The criterion for validation is as follows: when performing a two-stage classification for valence, the stage that yields the highest level of arithmetic mean of all folds in a four-fold cross validation is optimized. We used the same optimized parameter for 10-fold cross validation. The accuracy was computed by the equation TP/TP+FN using sensitivity analysis, where TP indicated true positives and FN indicated false negatives.

**Figure 7:**
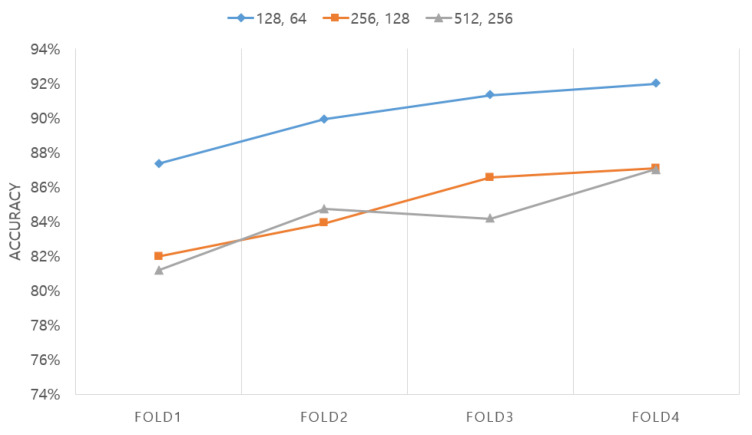
Comparison between accuracies obtained according to the number of cells in the LSTM layer.

First, as shown in Figure 7, the number of cells in the LSTM layer was optimized. In this case, the network was configured as an LSTM + attention network, as described in Section 2.3. The number of LSTM layers was fixed at two and the number of cells was tested three times: with 128 and 64 combinations, 256 and 128 combinations, and 512 and 256 combinations. The length of the EEG segment was fixed at 15 s. The experimental results showed that the highest accuracy of 90.14% was obtained for the layer with 128 and 64 combinations of cells, followed by the network with 256 and 128 combinations of cells (84.87%) and then the network with 512 and 256 combinations of cells (84.27%).

**Figure 8:**
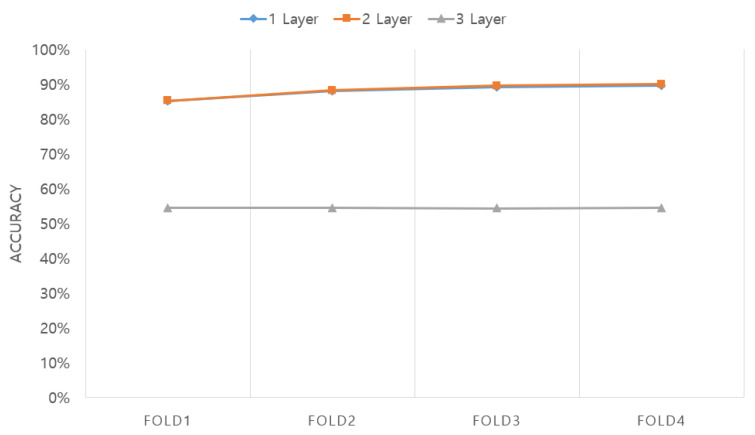
Accuracy obtained according to the number of LSTM layers.

The second optimized parameter in Figure 8 is the number of LSTM layers. In this case, the optimization was conducted using the configuration of the LSTM + attention network with one layer composed of 128 cells, two layers composed of 128 and 64 cells, and three layers composed of 128, 64, and 32 cells. The length of the EEG segment was fixed at 15 s, and the optimization result was 88.01%, 88.32%, and 54.59% in the case of one, two, and three layers, respectively. As is demonstrated, the accuracy was the highest in the case of two layers.

**Figure 9:**
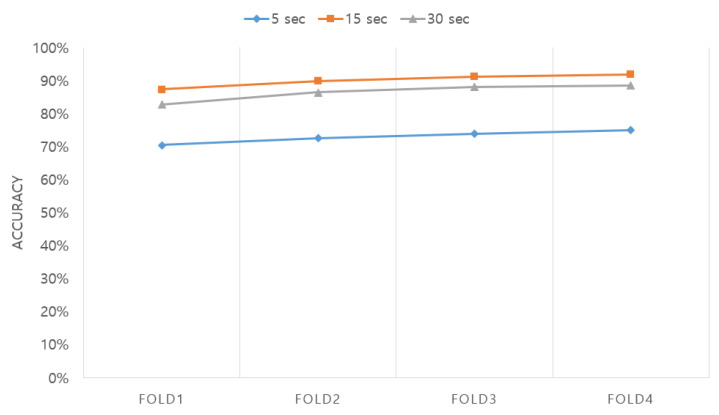
Accuracy in terms of different window sizes according to the length of the EEG segment.

Next, we optimized the length of the EEG segment as shown in Figure 9. The lengths of the windows used to segment the EEG were approximately 5, 15, and 30 s, which were exactly 5.25, 15.75, and 31.50 s, respectively. The network was a two-layer LSTM + attention network consisting of 128 and 64 cells. The optimization results showed accuracies of 73.01%, 90.14%, and 86.48% for the approximately 5, 15, and 30 s segments, respectively, clearly indicating that the 15 s segment shows the best accuracy. The result of parameter optimization showed that the optimal LSTM + attention network (Section 2.2) and LSTM + attention module (Section 2.3) are made of two LSTM layers consisting of 128 and 64 cells trained with 15 s EEG segments. The finally selected parameters are summarized in Table 1.

### 3.2. Experimental Results in Terms of LSTM and Attention Mechanism

**Figure 10:**
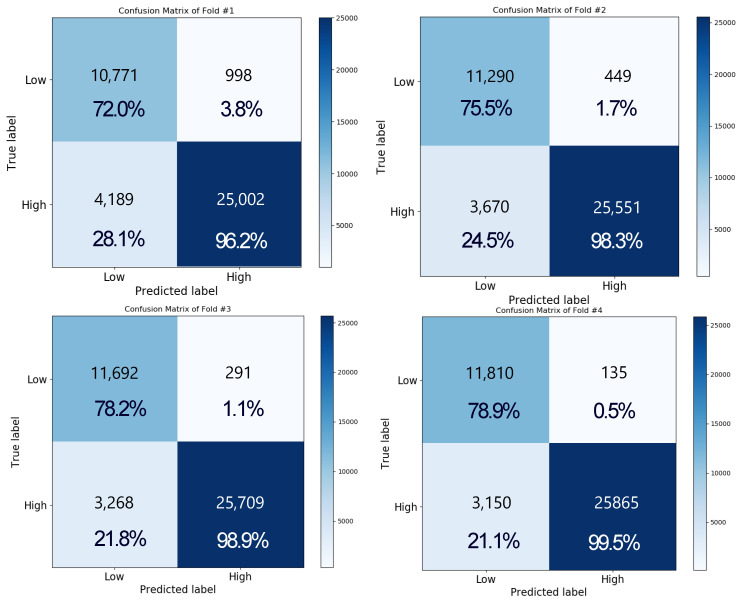
Confusion matrix representing the result of performing a two-level classification for valence by using the LSTM + attention network under the four-fold cross validation.

Figure 10 shows the result of performing a two-level classification for valence using the LSTM + attention network as a confusion matrix. For four-fold cross validation, the accuracies of folds 1, 2, 3, and 4 were 87.34%, 89.94%, 91.31%, and 91.98%, respectively, and the final accuracy was obtained as approximately 90.1 ± 2.05% by averaging the value of each fold. The accuracies for the low and high classes were obtained as approximately 76.1 ± 3.14% and 98.0 ± 1.45% on average, respectively. The overall recognition rate of the high class is higher than that of the low class. This is because SAM-labeled data in the DEAP database are not evenly distributed in high and low classes. The total number of low class data points used in the experiment is 14,960, and the total number of high class data points is 26,000. In the low class, on average, 23.85 ± 3.14% of the data points were low, but were misclassified as high class. In the high class, 1.80 ± 1.45% of the data points were high class, but were misclassified as low class. During training, high class data were about 1.7 times more than that of low class, so it was biased toward high class during training.

In the case of arousal recognition, the pattern was similar to that of valence. The accuracies of folds 1, 2, 3, and 4 were obtained as 84.62%, 87.61%, 89.41%, and 90.04%, respectively, and the final accuracy was approximately 87.9 ± 2.43%. Moreover, the matrix showed accuracies of approximately 76.27 ± 3.45% and 97.62 ± 1.59% for the low and high classes, respectively. The total number of low class data points used in the experiment is 18,600, and the total number of high class data points is 22,360. We checked the EEG signal when an error occurred, but we could not find any significant signal difference in the time domain. However, we observed that low class was incorrectly recognized as high class and high class as low class when there was a difference in the music video rating between subjects.

**Figure 11:**
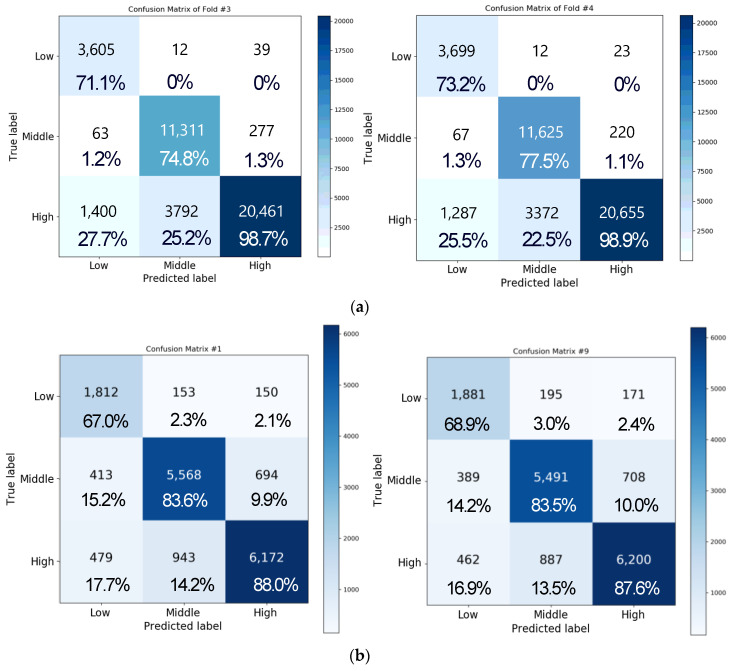
Confusion matrix representing the result of performing a three-level classification for valence by using LSTM + attention mechanism (**a**) with four-fold cross validation and (**b**) with 10-fold cross validation.

Figure 11a depicts the confusion matrix of the result of performing three-level classification for valence by using the same network as in the aforementioned experiment with four-fold cross validation. In this case, the accuracies of folds 1, 2, 3, and 4 were obtained as 77.24%, 82.61%, 86.37%, and 87.84%, respectively, and the average accuracy was approximately 83.51 ± 4.73%. Moreover, the matrix of each class showed accuracies of approximately 64.17 ± 9.45%, 72.39 ± 4.75%, and 96.2 ± 3.96% for the low, medium, and high classes, respectively. The data distribution for each class is as follows. There were 5017 data points for the low class, 14,976 data points for the medium class, and 20,967 data points for the high class in the one-fold verification. Most of the errors were caused by incorrectly classifying low class and medium class into high class. Since more than half of the data were of high class, the model was trained by biasing toward the high class, so the accuracy of the low class or medium class was lower than that of the high class. 

For arousal analysis of three-level classification, overall average accuracy was 82.57 ± 4.73%, and medium class showed the best performance compared to the other classes. The classes showed accuracies of 68.95 ± 7.40%, 95.78 ± 3.74%, and 73.11 ± 4.75% for the low, medium, and high classes, respectively. Unlike the valence analysis, we observed that the medium class showed the best classification result. As a result of analyzing the data of each class, we found that the number of data points was the highest with 18,320 in the medium class, 15,497 in the high class, and 7134 in the low class, and the data were reduced in the order of medium, high, and low. Therefore, like valence analysis, we observed that the accuracy increases with the amount of acquired data.

**Figure 12:**
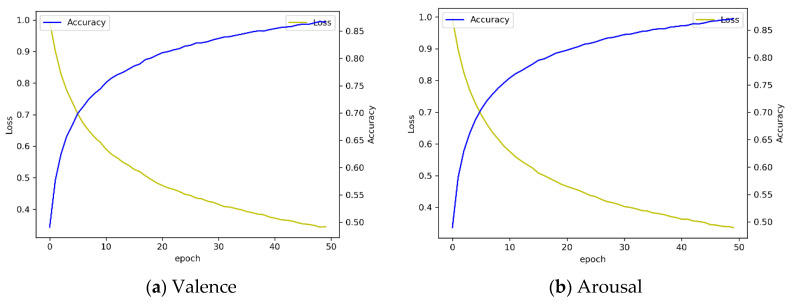
Loss and accuracy graph by using LSTM + attention mechanism with 10-fold cross validation.

Figure 11b illustrates the confusion matrix of three-level valence analysis with 10-fold cross validation. We observed that the overall accuracy was 82.31 ± 0.37% for valence. Accuracy for the low, medium, and high class was 68.14 ± 2.77%, 85.05 ± 1.66%, and 85.33 ± 2.51%, respectively. In addition, we observed that the overall accuracy was 81.71 ± 1.10% for arousal in three-level classification. Accuracy for the low, medium, and high class was 63.24 ± 5.20%, 85.68 ± 3.24%, and 85.30 ± 2.42%, respectively. Compared to four-fold cross validation and 10-fold cross validation, we found that the accuracy of 10-fold cross validation is lowered by about 1% when comparing the overall mean to four-fold cross validation. In the case of valence, the accuracy decreased by 1.2%, and in the case of arousal, the accuracy decreased by 0.86%. Through this result, we confirmed that there was no significant difference in the accuracy of 10-fold cross validation compared to four-fold cross validation through *t*-test-based statistical analysis (*p* > 0.05). Figure 12 shows the performance in terms of loss and accuracy as the number of epochs increases. We set the epoch parameter to 50 since there was a saturated result with 50 epochs.

### 3.3. Experimental Result by Using Attention LSTM and CNN Model

In this section, we explain the results of emotion classification using the LSTM + attention + CNN model. To compare the performance of the model, parameters, such as the number of LSTM layers, number of cells, length of the EEG segment, batch size, and epoch, were set identically in the main module comprising the LSTM + attention model. As EEG segments of the same length are used, the number of classes is the same as in Section 3.1. The two-level classification task using the network confirmed that the final accuracy was 90.1% for valence and 88.3% for arousal. Compared with the network using the LSTM with attention model, no improvement was observed in the valence accuracy and only 0.4% accuracy was observed for arousal. 

**Figure 13:**
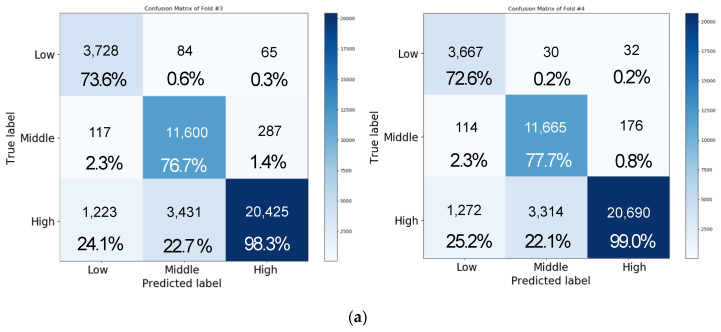

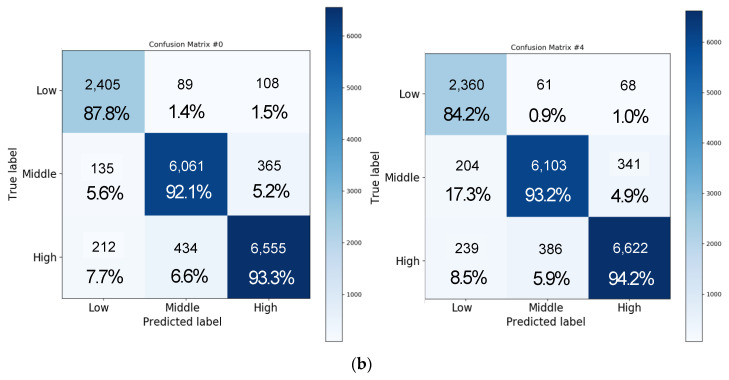
Confusion matrix representing the result of three-level classification for valence by using the LSTM + attention + CNN model (**a**) with four-fold cross validation and (**b**) with 10-fold cross validation.

Figure 13 shows the result of the three-level classification for valence when using the LSTM + attention + CNN model. Figure 13a illustrates the result of the three-level classification for valence with four-fold cross validation. The accuracies were obtained as 81.96%, 85.54%, 87.29%, and 87.94% in folds 1, 2, 3, and 4, respectively, with a final accuracy of 86.93 ± 1.24%. Moreover, the accuracies of each class, i.e., high, medium, and low classes, were 68.61 ± 5.57%, 74.42 ± 4.27%, and 97.91 ± 1.01%, respectively. In the case of valence analyzed at the three-level classification, 20,878 data points of the high class, 15,060 data points of the medium class, and 5022 data points of the low class were tested at a similar rate to the case of the two-level analysis. We also applied 10-fold cross validation to see if the current results can be generalized to a subject-independent model. Figure 13b shows that overall accuracy of three-level classification for valence was 91.77 ± 0.39%. The average accuracy for each class was 84.80 ± 1.68% for the low class, 92.81 ± 0.81% for the medium class, and 93.55 ± 0.59% for the high class for valence.

**Figure 14:**
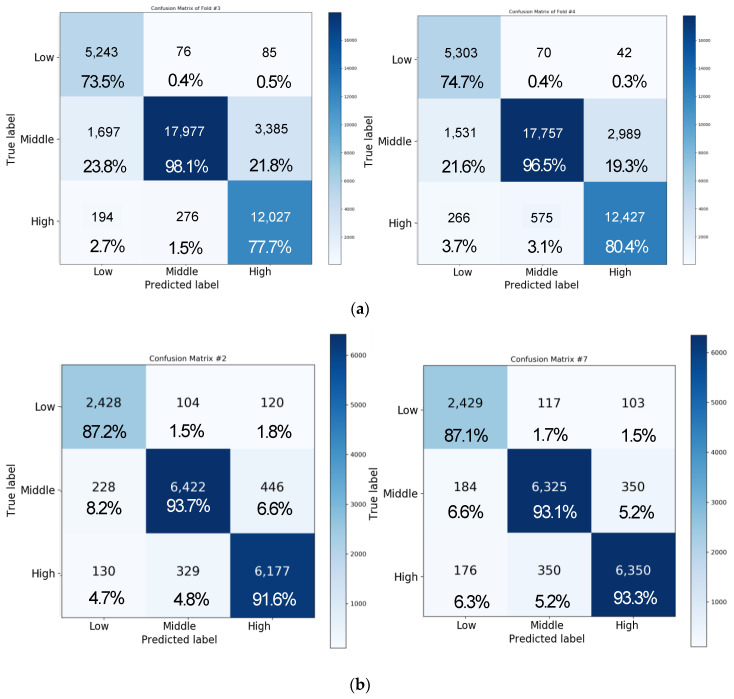
Confusion matrix representing the result of three-level classification for arousal by using the LSTM + attention + CNN model (**a**) with four-fold cross validation and (**b**) with 10-fold cross validation.

Figure 14 shows the result of three-level classification of arousal. As shown in Figure 14a, the accuracies with four-fold cross validation were obtained as 79.56%, 84.21%, 86.05%, and 86.64% in folds 1, 2, 3, and 4, respectively, with a final accuracy of 84.1 ± 3.21%. Moreover, the accuracies of each class, i.e., high, medium, and low classes, were 71.05 ± 4.55%, 92.2 ± 10.25%, and 80.6 ± 6.69%, respectively. The number of data points for each class of arousal was 7214 for low, 18,423 for medium, and 15,323 for high class. Therefore, unlike valence, there were a lot of data in the case of the medium class, which also affected the recognition performance, so the medium class performance was the best. Due to the nature of the emotional signal, it is easy to distinguish between positive and negative, but it is difficult to recognize the degree of arousal, such as severely aroused or less aroused, which affects the accuracy of arousal and valence. 

**Figure 15:**
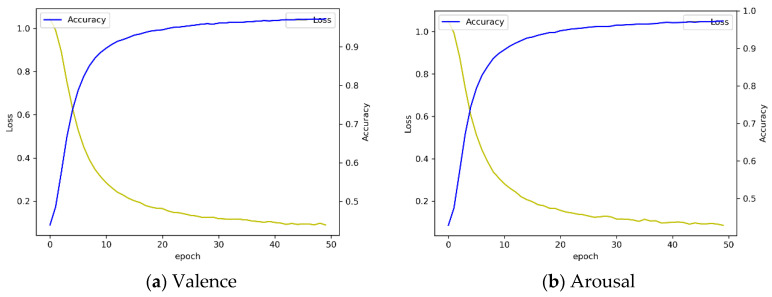
Loss and accuracy graph by using LSTM + attention + CNN model with 10-fold cross validation.

Figure 14b illustrates the result of three-level classification of arousal with 10-fold cross validation. The average accuracy of each 10-fold cross validation was 91.64 ± 0.34%. In the case of arousal, the accuracy of the low class was 82.28 ± 2.07%, the accuracy of the medium class was 92.78 ± 0.94%, and the accuracy of the high class was 93.39 ± 0.92%. Overall, the performance of the LSTM + attention + CNN model is improved with 10-fold compared to four-fold cross validation and, in particular, the medium class analysis result is greatly improved. In four-fold cross validation, the average accuracy of arousal was 84.1% and valence was 86.93%. In 10-fold cross validation, the accuracy of arousal improved by 7.54% and valence improved by 4.84% compared to four-fold cross validation. Overall loss and accuracy are shown in Figure 15. We observed that the loss in both valence and arousal analysis rapidly decreased after 10 epochs. The result was saturated at 50 epochs.

Table 2 compares the accuracy of the proposed method with existing two-level and three-level emotion classification methods with respect to arousal and valence analysis. As shown in Table 2, the accuracy of emotion recognition tends to decrease as the number of analyzed emotion recognition levels increases. The classification of the two classes with the attention LSTM showed an accuracy of 90.1% and 87.9% for valence analysis and arousal analysis under the four-fold cross validation method. The accuracy of the two-level classification emotion recognition in the previous studies was approximately 57.0–94.7% in terms of valance and 62.0–93.1% for arousal analysis [7,8,9,10,13,16,23,24,25]. The three-level emotion classification results were 53.4–60.7% for valance analysis and 46.0–62.33% for arousal analysis, which are lower than those obtained using the two-level classification [7,10,11]. Therefore, improving the emotion recognition results is necessary as the number of analyzed emotion recognition levels increases. However, in the performance comparison with previous studies, the validation method, amount of training/testing data, and parameter values to be used to train the model were different. In other words, since data collection, processing, and analysis were not performed in the same environment, it is difficult to draw conclusions only by comparing accuracy. Therefore, in the next section, Section 3.4, we analyzed the difference in performance according to the deep learning models in the same experimental environment.

### 3.4. Comparison with other Deep Learning Models

To verify the performance of the proposed network, we compared its accuracy with conventional deep leaning networks after setting the same input and parameter settings, as shown in Table 3. The comparative experiment was conducted using the LSTM excluding DNN, CNN, and attention mechanisms. Each network was configured as follows. The DNN consists of two dense layers, the output sizes of which were set to 200 and 100. The leaky ReLU activation function is applied to each dense layer after completing the operation. The CNN is the same as the structure in Figure 6; however, after passing through the 1-D convolution layer, its dimensions were reduced through max pooling. At this time, two convolution layers were used. For ≥3 layers, the accuracy did not increase compared to the learning time during parameter optimization and, therefore, the number of layers was set to two. LSTM removes the attention layer from the network proposed in Figure 2 and connects with the density layer after the second Bi-LSTM layer.

## 4. Discussion

In recent years, with the development of deep learning technology, many studies have been conducted to recognize emotions based on EEG signals. Existing emotion recognition technology based on EEG signals has the disadvantage that it is difficult to measure in everyday life, as well as difficult to use in general because the results are different depending on the researcher or the database used. The EEG signal has the advantage of being able to be measured noninvasively in everyday life. Headband-type devices have been recently released, and applications using EEG signals are being actively developed [10,34,35,36,37]. Therefore, in line with this, the development of a user-independent emotion recognition model that minimizes preprocesses such as feature extraction, noise filtering, and so on, and a model in which emotions are recognized robustly even when time changes, has laid the foundation for the application of emotion recognition technology to various fields. Based on these developments, emotion recognition technology has been introduced as a tool for communication with autistic children who cannot express their emotions by themselves [38,39]. In addition, recognized emotions can be used for brain–computer interaction [10] and for mental health treatment [40].

In this study, we investigated the end-to-end emotion recognition model while minimizing the preprocessing step to guarantee the emotion recognition accuracy in general. In addition, we proposed the model which supported more than two levels of emotion recognition and utilized the change in emotion recognition over time for improving emotion recognition accuracy. Therefore, we proposed two emotion classification models, one that applies an attention mechanism to LSTM and one that designed attention LSTM and a CNN in parallel. As a result of analysis through the experiment, we confirmed that in the case of the model applying a CNN to attention LSTM, there was no significant difference in performance in the two-level classification, but there was a performance improvement of about 1.6% to 1.7% in the three-level classification. Through this, we found that there is an effect when the number of classes to be classified increases when CNNs are applied in parallel under the same conditions. In the LSTM + attention model, the two-level and the three-level classification showed high performance in the high class, but the LSTM + attention + CNN model showed an improvement in the medium class. This indicated that the process of extracting features through CNNs is useful for EEG signal analysis. In the case of an EEG signal, it is a chaotic signal and noise can have a large influence due to external stimuli, so it is important to derive the characteristics of the signal itself. From this work, we observed that feature extraction through deep learning is useful compared to the case of using statistical features extracted from previous studies.

The experiments showed that the conventional methods succeeded in achieving approximately 94.7% performance accuracy in the two-level classification; however, for the three-level classification, they showed low and high accuracies of 46% and 62%, respectively. From this result, we found that the proposed LSTM with an attention mechanism showed performance improvement not only in two-level recognition but also in three-level recognition for recognizing emotions over time. In the three-level classification, the accuracy for the low class is approximately 4% and 25% lower than the accuracy for the middle and high classes, and this could be caused by data imbalance. These problems could be solved by applying data sampling and weight adjustment methods to achieve more accurate performances. In addition, we found that the valence prediction showed approximately 1–2% better results than the arousal prediction. A theory proposed by Howe et al. indicated that when a person remembers a word, he/she divides it into positive and negative aspects rather than by the level of positivity, and as such, remembers the word for a longer duration [40]. Therefore, we concluded that more distinct patterns are observed through deep learning because the score for valence is more objective than arousal.

As the level of emotion to be analyzed increases through the proposed method, the problem of a sharp drop in accuracy has been solved, but it is still challenging to analyze the level of emotion from 0 to 9. In order to solve this problem, it is necessary to design a fully connected layer at the last stage of the deep learning model or to study how to estimate emotions more accurately through regression. In addition, it is necessary to develop a subject-independent model in order to take into account differences according to people and measurement situations. Currently, raw EEG data have the advantage of minimizing data lost due to feature extraction, but EEG signals are chaotic signals and are generally analyzed in the frequency domain. Therefore, it is necessary to research and develop a model that can reflect the characteristics of the frequency domain well.

The limitations of this study are as follows. In the current experimental environment, training takes about 2 h, but there is a problem that the processing time linearly increases as the number of folds increases. Therefore, it is necessary to improve the performance in terms of time through model optimization. In addition, currently, the model has been verified and experimented with through the DEAP database, but similar performance should be guaranteed even when using a different type of EEG database or collecting and acquiring an actual EEG signal. In this study, the noise-filtered signal was applied as an input to recognize emotions through an end-to-end emotion model. However, in order to measure EEG in real life and analyze emotions through it, the emotion recognition model must be designed to be robust against noise. Therefore, as a future work, we plan to compare the results of well-processed and unprocessed input signals obtained in various environments, and conduct noise injection in the middle of the model layer or conduct a study on noise robustness through label-smoothing. Lastly, through existing neuroimaging studies, it can be seen that changes in emotion over time affect neurological brain activation, and these changes can appear in EEG signals over time [17,18,19]. However, it is not clear yet which channels of the EEG signal change according to brain activation, how the shape of the signal is reflected, and so on. Therefore, it needs to be verified through further research.

## 5. Conclusions

In this study, based on the peak–end rule theory, we proposed an LSTM deep learning model that effectively classifies valence and arousal dimensions with the application of an attention mechanism, which has recently gained research interest as a technology suitable for emotion analysis. The proposed model performs effective classification using only raw EEG signals without a separate feature extraction process. This method is expected to classify a person’s emotional state in real time in the field. The results show 90.1% and 87.9% accuracy, respectively, using a two-stage classification for the valence and arousal models with four-fold cross validation. For the three-stage classification with four-fold cross validation, these values were obtained as 83.5% and 82.6%, respectively. In 10-fold cross validation, we observed accuracy for valence was 82.31%, and accuracy for arousal was 81.71%. Further experiments were conducted using a network that combined the CNN submodule and the proposed model. The obtained results showed an accuracy of 90.1% and 88.3% in the case of the second-stage classification, and 86.9% and 84.1% in the case of the three-stage classification of the valence and arousal model with four-fold cross validation. In 10-fold cross validation, accuracy was 91.8% for valence and 91.6% for arousal.

As future research, we will apply the method to solve the aforementioned data imbalance by using weight balancing or regulation technology that can more effectively consider the EEG and emotion characteristics to predict more accurate emotional scores. In addition, it is necessary to improve accuracy with other databases or other EEG signals. Thus, we will collect the data with the same protocol, and need to check whether the proposed model can be used in a daily life. Then we will compare the results of well-processed and unprocessed input signals obtained from various data sources, and conduct noise injection in the middle of the model layer or conduct a study on noise robustness through label-smoothing. In addition, we will further research which channels of the EEG signal change according to brain activation, and how the shape of the signal is reflected.

## Figures and Tables

**Table 1:** Model parameters of the proposed method.

Category	Parameters	Numbers
Input	Length of EEG segment	15.75 s
	Batch size	32
	Epoch	30, 50
LSTM	Number of layers	2, bidirectional
	Number of cells	128, 64
	Number of dense layers	2
Attention	Number of attention layers	1
CNN	Number of layers	2
	Number of filters	128, 256
	Kernel size	64, 32
Training	Dropout probability	0.2
	Learning rate	0.001

**Table 2:** Results of two- and three-level classifications of conventional methods and proposed methods.

Research	Input	Method	Two-Level Classification		Three-Level Classification	
			Valence	Arousal	Valence	Arousal
Chung et al. [7]	Power spectral density, Power asymmetry	Bayes	66.6%	66.4%	53.4%	51.0%
Atkinson et al. [10]	Statistical features, Fractal dimension, Band power, Hjorth parameters	Support vector machine	73.1%	73.1%	60.7%	62.3%
Proposed method 1 *	Raw EEG signal	LSTM + Attention	90.1%	87.9%	83.5%	82.6%
Proposed method 2 *	Raw EEG signal	LSTM + Attention + CNN	90.1%	88.3%	86.9%	84.1%

* Four-fold cross validation case.

**Table 3:** Comparison of emotion classification accuracy between the proposed network and conventional deep learning networks.

Network	Two-Level Classification		Three-Level Classification	
	Valence	Arousal	Valence	Arousal
DNN	71.4%	61.6%	49.6%	42.2%
CNN	63.5%	54.6%	49.4%	43.4%
LSTM	89.7%	85.7%	50.9%	74.0%
Proposed method 1 * (LSTM + Attention)	90.1%	87.9%	83.5%	82.6%
Proposed method 2 * (LSTM + Attention + CNN)	90.1%	88.3%	86.9%	84.1%

* Four-fold cross validation case. DNN: Deep neural network, CNN: Convolutional neural network

## References

[1] Depression. https://www.who.int/news-room/fact-sheets/detail/depression.

[2] Huang X., Kortelainen J., Zhao G., Li X., Moilanen A., Seppänen T., Pietikäinen M. (2016). Multi-modal emotion analysis from facial expressions and electroencephalogram. Comput. Vis. Image Underst..

[3] Hemanth D.J., Anitha J., Son L.H. (2018). Brain signal based human emotion analysis by circular back propagation and Deep Kohonen Neural Networks. Comput. Electr. Eng..

[4] Al-Fahoum A.S., Al-Fraihat A.A. (2014). Methods of EEG Signal Features Extraction Using Linear Analysis in Frequency and Time-Frequency Domains. ISRN Neurosci..

[5] Phan K.L., Wager T., Taylor S.F., Liberzo I. (2002). Functional Neuroanatomy of Emotion: A Meta-Analysis of EmotionActivation Studies in PET and fMRI. NeuroImage.

[6] Lang P.J., Bradley M.M. (2010). Emotion and the motivational brain. Biol. Psychol..

[7] Chung S.Y., Yoon H.J. Affective classification using Bayesian classifier and supervised learning. Proceedings of the 2012 12th International Conference on Control, Automation and Systems.

[8] Koelstra S., Mühl C., Soleymani M., Lee J.S., Yazdani A., Ebrahimi T., Pun T., Nijholt A., Patras I. (2012). DEAP: A database for emotion analysis; Using physiological signals. IEEE Trans. Affect. Comput..

[9] Zhang X., Hu B., Chen J., Moore P. (2013). Ontology-based context modeling for emotion recognition in an intelligent web. World Wide Web.

[10] Atkinson J., Campos D. (2016). Improving BCI-based emotion recognition by combining EEG feature selection and kernel classifiers. Expert Syst. Appl..

[11] Jirayucharoensak S., Pan-Ngum S., Israsena P. (2014). EEG-Based Emotion Recognition Using Deep Learning Network with Principal Component Based Covariate Shift Adaptation. Sci. World J..

[12] Posner J., Russell J.A., Peterson B.S. (2005). The circumplex model of affect: An integrative approach to affective neuroscience, cognitive development, and psychopathology. Dev. Psychopathol..

[13] Al-Nafjan A., Hosny M., Al-Wabil A., Al-Ohali Y. (2017). Classification of Human Emotions from Electroencephalogram (EEG) Signal using Deep Neural Network. Int. J. Adv. Comput. Sci. Appl..

[14] Krishna N.M., Sekaran K., Vamsi A.V.N., Ghantasala G.S.P., Chandana P., Kadry S., Blazauskas T., Damaševičius R., Kaushik S. (2019). An efficient mixture model approach in brain-machine interface systems for extracting the psychological status of mentally impaired persons using EEG signals. IEEE Access.

[15] Karim F., Majumdar S., Darabi H., Harford S. (2019). Multivariate LSTM-FCNs for time series classification. Neural Netw..

[16] Yang H., Han J., Min K. (2019). A Multi-Column CNN Model for Emotion Recognition from EEG Signals. Sensors.

[17] Goldin P.R., Hutcherson C.A., Ochsner K.N., Glover G.H., Gabrieli J.D.E., Gross J.J. (2005). The neural bases of amusement and sadness: A comparison of block contrast and subject-specific emotion intensity regression approaches. NeuroImage.

[18] Raz G., Jacob Y., Gonen T., Winetraub Y., Flash T., Soreq E., Hendler T. (2014). Cry for her or cry with her: Context-dependent dissociation of two modes of cinematic empathy reflected in network cohesion dynamics. Soc. Cogn. Affect. Neurosci..

[19] Résibois M., Verduyn P., Delaveau P., Rotgé J., Kuppens P., Mechelen I.V., Fossati P. (2017). The neural basis of emotions varies over time: Different regions go with onset- and offset-bound processes underlying emotion intensity. Soc. Cogn. Affect. Neurosci..

[20] Zheng W.L., Zhu J.Y., Lu B.L. (2019). Identifying Stable Patterns over Time for Emotion Recognition from EEG. IEEE Trans. Affect. Comput..

[21] Hochreiter S., Schmidhuber J. (1997). Long Short-Term Memory. Neural Comput..

[22] Huang C.W., Narayanan S. Deep convolutional recurrent neural network with attention mechanism for robust speech emotion recognition. Proceedings of the IEEE International Conference on Multimedia and Expo (ICME).

[23] Alhagry S., Aly A., El-Khoribi R. (2017). Emotion Recognition based on EEG using LSTM Recurrent Neural Network. Int. J. Adv. Comput. Sci. Appl..

[24] Anubhav A., Nath D., Singh M., Sethia D., Kalra D., Indu S. An Efficient Approach to EEG-Based Emotion Recognition using LSTM Network. Proceedings of the 16th IEEE International Colloquium on Signal Processing & Its Applications (CSPA 2020).

[25] Xing X., Li Z., Xu T., Shu L., Hu B., Xu X. (2019). SAE+LSTM: A New framework for emotion recognition from multi-channel EEG. Front. Nuerorobot..

[26] Fredrickson B.L., Kahneman D. (1993). Duration Neglect in Retrospective Evaluations of Affective Episodes. J. Pers. Soc. Psychol..

[27] Vaswani A., Shazeer N., Parmar N., Uszkoreit J., Jones L., Gomez A.N., Kaiser L., Polosukhin I. Attention Is All You Need. Proceedings of the 31st Conference on Neural Information Processing Systems (NIPS 2017).

[28] Yao Y., Zhang S., Yang S., Gu G. (2020). Learning Attention Representation with a Multi-Scale CNN for Gear Fault Diagnosis under Different Working Conditions. Sensors.

[29] Bello I., Zoph B., Vaswani A., Shlens J., Le Q.V. Attention Augmented Convolutional Networks. Proceedings of the IEEE/CVF International Conference on Computer Vision (ICCV).

[30] Fukui H., Hirakawa T., Yamashita T., Fujiyoshi H. Attention Branch Network: Learning of Attention Mechanism for Visual Explanation. Proceedings of the IEEE/CVF Conference on Computer Vision and Pattern Recognition (CVPR).

[31] Mirsamadi S., Barsoum E., Zhang C. Automatic speech emotion recognition using recurrent neural networks with local attention. Proceedings of the 2017 IEEE International Conference on Acoustics, Speech and Signal Processing (ICASSP).

[32] Li P., Yan S., McLoughlin I., Guo W., Dai L. An Attention Pooling based Representation Learning Method for Speech Emotion Recognition. Proceedings of the International Speech Communication Association.

[33] Schuster M., Paliwal K.K. (1997). Bidirectional recurrent neural networks. IEEE Trans. Signal Process..

[34] Casson A.J., Yates D.C., Smith S., Duncan J.S., Rodriguez-Villegas E. (2010). Wearable electroencephalography. IEEE Eng. Med. Biol. Mag..

[35] Lin C.T., Chuang C.H., Huang C.S., Tsai S.F., Lu S.W., Chen Y.H., Ko L.W. (2014). Wireless and Wearable EEG System for Evaluating Driver Vigilance. IEEE Trans. Biomed. Circuits Syst..

[36] Brainbit. https://brainbit.com/.

[37] Neurosky. http://neurosky.com/.

[38] Picard R.W. (2009). Future affective technology for autism and emotion communication. Philos. Trans. R. Soc. B Biol. Sci..

[39] Howe M.L., Candel I., Otgaar H., Malone C., Wimmer M.C. (2010). Valence and the development of immediate and long-term false memory illusions. Memory.

[40] Liu Y., Sourina O., Nguyen M.K. (2011). Real-Time EEG-Based Emotion Recognition and Its Applications. Trans. Comput. Sci. XII LNCS.

